# KDM5A overexpression modulates key myogenic differentiation factors and sarcomere gene expression

**DOI:** 10.17912/micropub.biology.002136

**Published:** 2026-10-07

**Authors:** Geert de Vries, Alice Monticelli, Vicky De Winter, Danique Beijer, Willem De Ridder, Jonathan Baets

**Affiliations:** 1 Translational Neurosciences and Peripheral Neuropathy Group, UAntwerp, Antwerp, VLG, BE; 2 Laboratory of Neuromuscular Pathology, Institute Born-Bunge, UAntwerp, Antwerp, VLG, BE; 3 Peripheral Neuropathy Research Group, Department of Biomedical Sciences, UAntwerp, Antwerp, VLG, BE; 4 Department of Neurology, Neuromuscular Reference Centre, Antwerp UH, Antwerp, VLG, BE

## Abstract

Myogenic differentiation is a complex and tightly regulated process involving multiple transcriptional regulators. KDM5A (lysine demethylase 5A) is a histone demethylase that regulates gene transcription and has been implicated in skeletal muscle differentiation. In Inclusion Body Myositis, we previously associated dysregulated myogenic differentiation with KDM5A overactivity. Immortalised human myoblasts overexpressing KDM5A showed an altered transcriptional landscape, with differential expression of key myogenic differentiation factors and significantly increased expression of sarcomeric genes. Although no morphological differences were observed, KDM5A-overexpressing myotubes showed a higher proportion of myogenin-positive nuclei during late-stage differentiation, suggesting that KDM5A modulates myogenic differentiation dynamics.

**Figure 1. Overexpression of KDM5A in immortalised myoblasts results in modulation of the transcriptional landscape of in vitro myogenic differentiation and increases differentiation activity in the absence of morphological aberrations:** **A)** Representative images of immunofluorescent staining of MYH2 (yellow) and nuclear staining with Hoechst 33342 (blue) in differentiated myotubes at day 7 of *in vitro* differentiation of *KDM5A* -overexpressing and *GFP* -control myoblasts. Scale bar: 50 μm. **B)** Quantification of fusion index, to evaluate differentiation efficiency, and myotube area, as area of myosin heavy chain positive cell area as percentage of the total image. Wilcoxon signed rank test was applied on n=3 independent biological replicates **C)** Representative images of immunofluorescent staining of myogenin (yellow) and nuclear staining with Hoechst 33342 (blue) in differentiated myotubes at days 0, 3 and 7. Scale bar overview and zoom: 50 μm. **D)** Quantification of myogenin-positive nuclei, as percentage of the total number of nuclei segmented, during *in vitro* differentiation. **E)** Representative western blot probed for MYH2, myogenin, and a-tubulin as a loading control at differentiation days 0, 3, and 7. **F)** Densitometric quantifications of protein expression, normalised to a-tubulin, demonstrating significantly increased MYH2 abundance at day 7 upon KDM5A overexpression, while myogenin levels are similar. **G-I)** Fold change quantifications of qRT-PCRs at days 0, 3, and 7 of *in vitro* differentiation, targeting **G)** myogenic regulatory factors *Myf5* , *MYOD1* , *myogenin,* and *MRF4* , **H)** myosin heavy chain isoforms *MYH2* , *MYH3* , and *MYH8* , and **I)** maturation markers *TNNI2* and *CKM* . A two-way mixed model ANOVA with Sidak’s post hoc test was applied for comparisons across time points and cell lines on n=3 independent biological replicates. For all graphs, data presented as mean ± s.d.;. ns, not significant; *** p<0.0001; ** p<0.001; * p<0.05

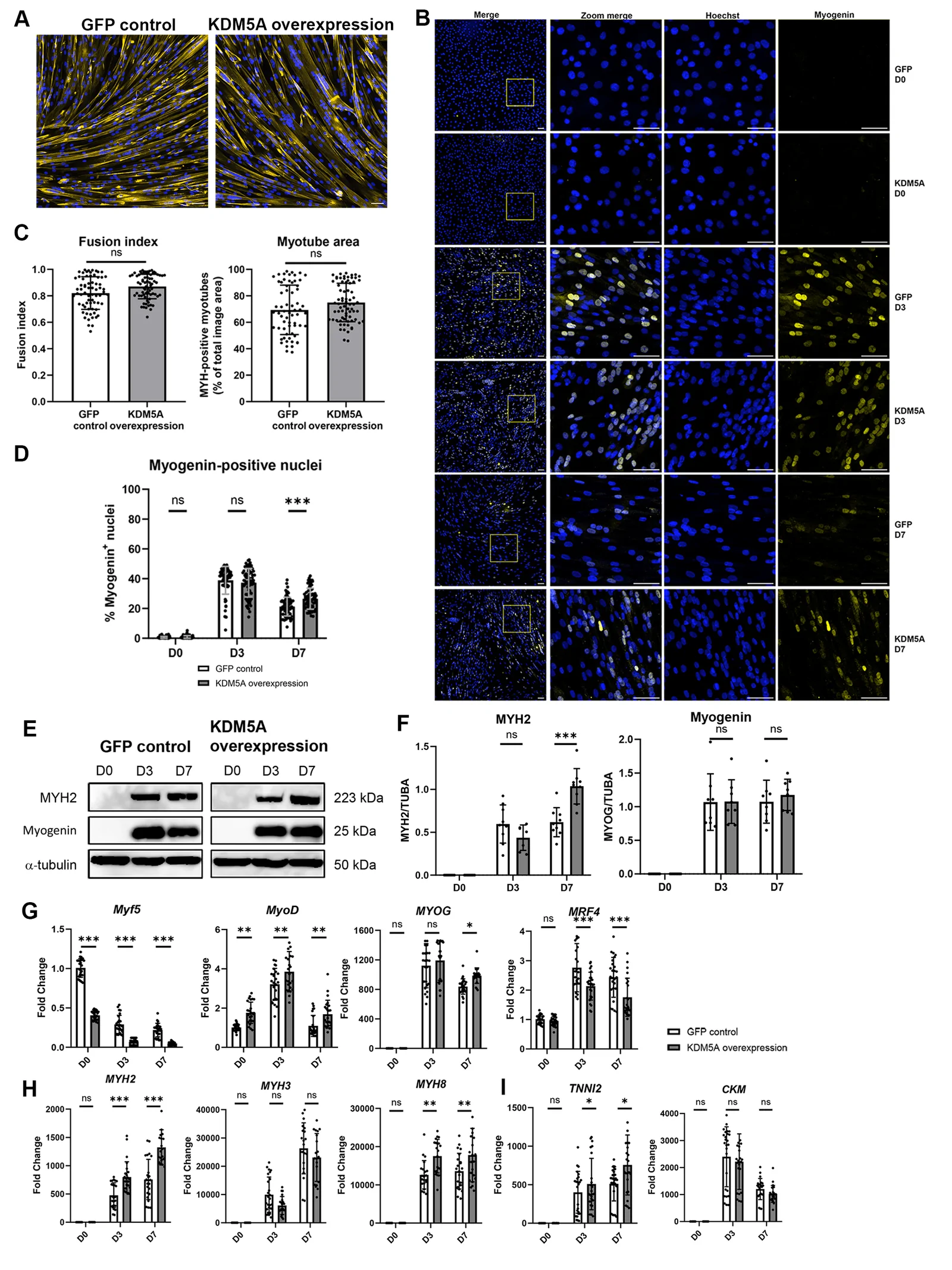



## Description

Myogenic differentiation in skeletal muscle is a complex and tightly regulated process, orchestrated by four major myogenic regulatory factors (MRFs): Myf5, MyoD *(MYOD1),* myogenin ( *MYOG)* , and MRF4.(Ancel et al., 2021; Zammit, 2017) Functioning in a temporally ordered manner, each MRF has distinct roles in the different stages of commitment, differentiation, and maturation of myogenic precursor cells into mature myotubes.(Bentzinger et al., 2012; Zammit, 2017) Perturbation of the regulation underlying myogenic differentiation negatively impacts regeneration of myofibres and can lead to chronic muscle wasting.(Dell’Orso et al., 2019; Hou et al., 2025) Chronic and progressive muscle wasting is the hallmark of a large number of myopathies, leading eventually to sarcopenia and ultimately loss of ambulation.(Benveniste et al., 2011; Dalakas, 2023) This is particularly evident in Inclusion Body Myositis (IBM), an acquired myopathy characterised by dual inflammatory-degenerative pathology which leads to progressive and currently untreatable muscle wasting.(Greenberg, 2019; Lundberg et al., 2021) Paradoxically, IBM patients display increased regeneration under these pathological conditions, while simultaneously displaying progressive muscle atrophy.(de Vries et al., 2025; Wanschitz et al., 2013)

Previously, we identified KDM5A overactivity as potential upstream driver of IBM pathology, with a possible role in the dysregulation of myogenic differentiation.(de Vries et al., 2025) KDM5A is a strictly nuclear histone demethylase involved in transcriptional regulation of genes involved in cellular processes including proliferation and differentiation, and is described as key regulator of myogenic differentiation through the expression of mitochondrial and contractile proteins.(Benevolenskaya et al., 2005; de Vries et al., 2025; Váraljai et al., 2015) Here, we aimed to investigate the effects of KDM5A overexpression on *in vitro* myogenic differentiation by evaluating its regulatory impact on the expression of key myogenic differentiation factors and maturation-associated proteins.

A KDM5A-overexpressing immortalised human myoblast line was generated using a plasmid-based expression system, with overexpression validated using qRT-PCR and western blot, demonstrating clear and significant overexpression maintained throughout the whole differentiation period (Extended data Figure S1).

Evaluating myogenic differentiation on a morphological level, we observed no overt differences or aberrations ( Fig. 1A ). Differentiation efficiency was quantified with the fusion index, which showed a near-significantly higher level (p=0.08) in the KDM5A overexpression line ( Fig. 1B ). In addition, no significant differences in the area covered by all myotubes per field of view were observed. As we previously observed an increased percentage of myogenin-positive nuclei in patient-derived IBM muscle tissue, we stained for myogenin in differentiating myotubes ( Fig. 1C ).(de Vries et al., 2025) Here, we observed only at differentiation day 7 a higher percentage of myogenin-positive nuclei (p<0.0001) upon KDM5A overexpression (Fig 1D).

We next examined these key differentiation markers, myogenin and myosin heavy chain 2 (MYH2), at the protein level ( Fig. 1E ). Showing the expected differentiation dynamics, MYH2 peaked at differentiation day 7, while myogenin remained relatively stable over time ( Fig. 1F ). In KDM5A-overexpressing myotubes, MYH2 protein abundance was significantly increased at late-stage differentiation compared with controls (p<0.0001), whereas no significant differences were observed in myogenin protein levels ( Fig. 1F ).

With KDM5A described as a transcriptional regulator of myogenic differentiation, we evaluated with qRT-PCR the expression of the major MRFs and sarcomeric proteins expressed during *in vitro* differentiation. Among the MRFs, we found significantly decreased *Myf5* levels (all p<0.0001) and increased *MYOD1* levels (all p<0.01) across all differentiation time points upon KDM5A overexpression ( Fig. 1G ). *MYOG* expression was significantly increased at differentiation day 7 (p=0.011). Conversely, *MRF4* , typically co-expressed with myogenin during late-stage differentiation, showed significantly reduced expression in KDM5A-overexpressing myotubes at days 3 (p=0.0003) and 7 (p<0.0001).

To evaluate myotube development and maturation, we examined the expression of myosin heavy chain isoforms *MYH2* , *MYH3* , and *MYH8* . At differentiation day 3 and 7, KDM5A-overexpressing myotubes exhibited significantly higher levels of *MYH8* (p=0.0009 and p=0.004) and *MYH2* (both p<0.0001), whereas the decreased expression of *MYH3* at these time points was not statistically significant ( Fig. 1H ). Other markers of myotube maturation, *TNNI2* and *CKM* , increased as expected with differentiation. *TNNI2* showed variable but significantly higher expression in KDM5A-overexpressing myotubes at days 3 (p=0.024) and 7 (p=0.011). Differences in CKM expression did not reach statistical significance ( Fig. 1I ).

Previously, we identified KDM5A overactivity as a potential driver of IBM pathology with a possible specific involvement in the dysregulation of myogenic differentiation.(de Vries et al., 2025) Here, we present the first data on the downstream effects of overexpression of KDM5A, a histone demethylase, on *in vitro* myogenic differentiation in immortalised human myoblasts. Our results show KDM5A overexpression differentially regulates key myogenic regulatory factors (MRFs) and increases the expression of maturation- and sarcomere-associated genes, particularly during late-stage differentiation. Together, these findings indicate that KDM5A modulates the myogenic differentiation program and suggest increased differentiation and maturation activity at the transcriptional level. Interestingly, these molecular changes were not accompanied by overt morphological alterations, as no significant differences in fusion index or total myotube area were observed compared with GFP controls.

As myogenic differentiation is a tightly regulated process, the differential expression of the MRFs resulting from KDM5A overexpression demonstrates that the modest effect sizes as we observed facilitate more global alterations in the regulation of *in vitro* myogenic differentiation. In particular, the altered expression of the early stage MRFs, *Myf5* and *MYOD1* , might reflect increased commitment of myoblasts towards differentiation rather than proliferation.(Kitzmann & Fernandez, 2001) In addition, the higher percentage of myogenin-positive nuclei in late-stage differentiation, consistent with our previous findings in IBM muscle, together with the differential expression of later stage markers *MYOG* and *MRF4* , further supports the notion of increased differentiation activity and myotube maturation.(de Vries et al., 2025; Hinterberger et al., 1991; Zhang et al., 1995) Through its effects on myogenic differentiation, KDM5A may potentially drive the increased regenerative response observed in IBM muscle tissue and thereby contribute to the dysregulation of the differentiation process.

Investigating the precise mechanisms through which KDM5A overexpression differentially regulates the genes involved in myogenic differentiation was beyond the scope of this study. KDM5A has been relatively little studied as a regulator of myogenic differentiation, with only limited data available on its role in this context. One previous study investigating KDM5A overactivity in transdifferentiated myoblasts found KDM5A overactivity, induced through Rb1 knockout, represses mitochondrial gene expression, resulting in dysfunctional differentiation *in vitro* .(Váraljai et al., 2015) An important methodological distinction here is that we induced KDM5A overactivity through KDM5A overexpression directly using viral transduction, whereas the previous study achieved increased KDM5A activity through Rb1 knockout, an established KDM5A interaction partner.(Benevolenskaya et al., 2005) Notably, reduced Rb1 function, through knockout or knockdown, has been described in several studies to negatively impact myogenic differentiation.(Ciavarra & Zacksenhaus, 2010; Kohno et al., 2025; Váraljai et al., 2015) As such, the mechanism through which KDM5A overexpression results in increased myogenic differentiation activity may depend on cooperation with Rb1. In turn, this directly alters the transcriptional activity of the MRFs, thereby modulating the downstream myogenic transcriptional program and resulting in increased differentiation activity.

Limitations of this study relate primarily to the relative sensitivity and methodological thresholds of the different assays applied as we controlled for experimental biases such as differences in expression timing and cellular heterogeneity by optimising the experimental setup. The intrinsic constraints of the techniques employed potentially precluded detection of subtle effects or small effect sizes. This may be particularly the case with evaluating myogenin, where statistically significant differences detected at the transcriptional level and with immunofluorescence were not reflected in bulk protein measurement, underscoring the importance of combining complementary techniques to capture nuanced, late-stage differentiation effects.

Our work provides a concise yet detailed and literature-aligned profiling of the major and most commonly used markers of *in vitro* myogenic differentiation as influenced by KDM5A overexpression.(Stern-Straeter et al., 2011; VanGenderen et al., 2022) Future directions include a focus on the broader transcriptional alterations induced by KDM5A overexpression during myogenic differentiation, especially in the myoblast stage where we observed significant differential expression of the MRFs in KDM5A-overexpressing myoblasts. These transcriptional alterations may indicate differences in proliferation, priming, and commitment to differentiation, thereby potentially playing a role in increased differentiation activity downstream of KDM5A overactivity.(Kitzmann & Fernandez, 2001) In that context, the upregulation of myogenin by KDM5A observed here is particularly interesting as this may be relevant in other myopathies that display repeated degeneration-regeneration cycles (e.g. muscular dystrophies) or regeneration in an inflammatory environment (e.g. IBM).(Deprez et al., 2023; Dumont et al., 2015)

In summary, KDM5A overexpression alters the transcriptional landscape of *in vitro* myogenic differentiation through differential regulation of the major MRF genes, resulting in increased downstream differentiation activity and sarcomeric gene expression particularly during late-stage canonical *in vitro* myogenic differentiation. These results further support the involvement of KDM5A overactivity in dysregulation of myogenic differentiation in IBM as it potentially drives the increased regenerative activity observed in IBM. Further mechanistic studies are warranted to determine how KDM5A interacts with the myogenic regulatory network and whether these effects contribute to the pathological muscle regeneration observed in IBM. Such studies may provide broader insights into dysregulated myogenic differentiation in IBM and other myopathies in which impaired muscle regeneration is a prominent feature.

## Methods

*Cell culture*

Human myoblasts isolated from muscle from a 53-years-old healthy man and immortalized as previously described, were provided anonymously by MYOBANK (made available through collaboration by Prof. Vincent Mouly), a tissue bank affiliated with EUROBIOBANK.(de Vries et al., 2025; Mamchaoui et al., 2011) Human myoblasts were cultured in Skeletal Muscle Cell Medium (PromoCell; C-23060) at 37°C in a humidified atmosphere containing 5% CO _2_ . Myoblasts were differentiated to myotubes in DMEM+Glutamax high glucose (Life Sciences; 11574516) supplemented with 2% horse serum (Life Technologies; 26050070) and 1% penicillin-streptomycin (Life Technologies; 15140122) in geltrex-coated (ThermoFisher; A1313301) Ibidi 96-well plates (Ibidi; 89626) for immunostainings or on geltrex-coated tissue culture plates for western blot or qRT-PCR samples. Myogenic differentiation was assessed by sampling at three time points (day 0, 3, and 7) during *in vitro* differentiation. Cell pellets were shock-frozen in liquid nitrogen and stored at -80 °C. In parallel, geltrex-coated 96-well Ibidi plates were fixed at all time points to evaluate myogenin-stained nuclei and at day 7 to assess differentiation efficiency.

*Generation overexpression cell lines*

Human myoblasts stably overexpressing either *KDM5A* or GFP-vector were generated through lentiviral transduction of the healthy control myoblast cell line. HEK293T cells were transiently transfected using linear polyethylenimime PEI MAX 40K (24765-1, PolySciences Europe), with packaging (pCMV dR8.91), envelope (pMD2-VSV) and custom Lenti-hCMV-KDM5A-eGFP-IRES-BSD plasmid (Transomic Technologies), containing the open reading frame of *KDM5A* (GenBank accession number BC156461), or a previously generated GFP-pLenti6/V5-DEST (Life Technologies, V49610) in DMEM+Glutamax high glucose (Life Sciences; 11574516) supplemented with 10% FBS.(Haidar et al., 2019) After 48h, the virus containing supernatant was collected from the HEK293T cells, filtered through a 0.45 μm filter (SLHV033RB, Millipore) and added to the Skeletal Muscle Cell Medium to infect the immortalised myoblasts. Virus medium was removed after 24h and replaced by fresh medium. Selection for infected cells was carried out by adding blasticidin S (5 μg/ml; ant-bl-1, InvivoGen Europe). Cells were cultured at 37 °C and 5% CO _2_ .

*Immunocytochemistry and image analysis*

Cultured cells were fixed with 4% paraformaldehyde in PBS for 20 min at room temperature, washed three times with PBS and stored at 4°C until staining. Cells were incubated with blocking buffer, containing goat serum (Jackson Immunoresearch; 005-000-121) 1:500 in PBT (PBS with 0.5% BSA and 0.5% Triton-X-100) for 1h at room temperature, followed by incubation with anti-myosin heavy chain 2 (MYH2) (1:1000 in PBT) or anti-myogenin (1:1000 in PBT) for 2h. Wells were washed three times with PBS prior to secondary antibody incubation. This was performed with goat-anti-mouse-AlexaFluor594 conjugated secondary antibody (1:500 in PBT) for 1h at room temperature, followed by three more washes with PBS. Nuclear staining was performed with Hoechst (1:20,000 in PBT) for 10 min at room temperature, followed by three washes with PBS.

Image z-stacks were acquired with a Nikon Ti2 microscope using a 20x/0.8 Plan Apochromat objective. Per well, 8 random positions were automatically imaged using the JOBS module of Nikon NIS Elements software. Image z-stacks were converted to maximum intensity projections and analysed with CellProfiler 4.2.8 (Cimini Lab, Broad Institute) to assess differentiation efficiency by quantifying the fusion index and to calculate the number of Myogenin-positive nuclei as proportion of the total number of nuclei per field of view.(Stirling et al., 2021)(Stirling et al., 2021) Fusion index was defined here as the number of nuclei in MYH2-positive (MYH2 ^+^ ) myotubes divided by the total number of nuclei segmented.(VanGenderen et al., 2022)

*Western blot*

Myoblast pellets were lysed in RIPA buffer with 0.5% SDS. Soluble protein extracts were obtained after centrifugation. Protein quantification was performed with the Pierce BCA assay kit according to manufacturer’s instructions (ThermoFisher; 23227). 15 μg protein was loaded and separated on 4–12% NuPAGE ^®^ Bis-Tris gels (Life Technologies; 10247002) and transferred on Protran Premium 0.45 µm nitrocellulose membranes (Amersham Biosciences; 10600003). Membranes were incubated overnight at 4°C with primary antibodies (listed Supplementary Table 1), all at a dilution of 1:1000 in 5% skimmed milk powder in PBS-T (PBS with 0.1% Tween-20). Immunodetection was performed using host-specific HRP-conjugated secondary antibodies (Supplementary Table S1). SuperSignal™ West Femto Maximum Sensitivity Substrate (ThermoFisher Scientific; 34096) for KDM5A and myogenin and Pierce™ ECL Plus western Blotting Substrate (ThermoFisher Scientific; 32132) for MYH2 and a-tubulin were used for visualisation on the Amersham™ Imager 680 digital imaging system (GE Healthcare). Densitometric analysis was performed using the Fiji distribution of ImageJ v1.54p, with each target normalised to α-tubulin abundance for statistical analysis.(Schindelin et al., 2012)

*Quantitative Reverse Transcription PCR (qRT-PCR)*

For qRT-PCR, RNA was isolated from samples harvested and snap frozen at different time points of differentiation. Total RNA was isolated using the Roboklon Universal RNA isolation kit (Roboklon GmbH; E3598-02) and cDNA was synthesised with the High-Capacity Reverse Transcription kit using 250 ng RNA total input, further following manufacturer’s instructions (ThermoFisher; 4368814). Primers were designed using Primer3web software, with efficiency assessed for all primers used (Supplementary Table S2).(Quellhorst & Rulli, 2012; Untergasser et al., 2012) qRT-PCR was carried out on a QuantStudio 6 Flex machine with Viia6 software (Applied Biosystems). Amplification was performed with the following parameters: (1x) 50°C: 2 min, 95°C: 10 min, (40x) 95°C: 15 sec, 60°C: 1 min, melt curve. Data analysis was carried out using the 2 ^-DDCt^ method to calculate fold changes, with genes of interest normalised to Ribosomal Protein Lateral Stalk Subunit P0 (RPLP0) and TATA-Box Binding Protein (TBP).(Stern-Straeter et al., 2009)

*Statistical analysis*

For all read-outs, data from three independent experiments, each including three technical replicates, were pooled and analysed using GraphPad Prism 8.0.1 (GraphPad software) for data visualisation and statistical testing. A two-way mixed model ANOVA with Sidak’s post hoc test was applied for comparisons across time points and cell lines. For statistically testing the fusion index, a Wilcoxon signed rank test was applied. A p-value <0.05 was considered statistically significant.

## Reagents

**Table:** 

**Antibodies**	**Manufacturer; catalogue number**	**Species; clonality**	**Application**
*Primary antibodies*			
Myosin heavy chain 2	Merck; M2476	Mouse; monoclonal	western blot, ICC
Alpha-tubulin	Abcam; ab7291	Mouse; monoclonal	western blot
Myogenin	Abcam; ab1835	Mouse; monoclonal	western blot, ICC
KDM5A	ThermoFisher; MA5-34682	Rabbit; monoclonal	western blot
			
*Secondary antibodies*			
Goat-anti-Rabbit HRP-conjugated	Jackson ImmunoResearch; 111-035-144	Goat; polyclonal	western blot
Goat-anti-Mouse HRP-conjugated	Jackson ImmunoResearch; 115-035-146	Goat; polyclonal	western blot
Goat-anti-Mouse(IgG1) HRP conjugated	Southern BioTech; 1070-05	Goat; polyclonal	western blot
Goat-anti-mouse AlexaFluor594	LifeTechnologies; A11032	Goat; polyclonal	ICC
Goat-anti-mouse(IgG1) AlexaFluor594	LifeTechnologies; A21125	Goat; polyclonal	ICC
			
**Target**	**Forward qRT-PCR primer**	**Reverse qRT-PCR primer**	
CKM	CTGACAAGCACAAGACTGACC	GCTGAGCACGTAGTTAGGG	
KDM5A	CAGAGTGAGATTGGATTTCTTG	GGTGACCATTTCAAAACCTCCT	
MRF4/Myf6	CCCTTCAGCTACAGACCCAAAC	CCCTGGAATGATCGGAAACA	
Myf5	CAGTCCTGTCTGGTCCAGAAAG	GTCCACTATGTTGGATAAGCAATC	
MYH2	AAGGTCTCCATTTACAAGCTCACG	TTGGACACCTGTTCTACAGTCTGG	
MYH3	TTGCTTCGTGGTGGACTCAA	CCATGTCTTCGATCCTGTCG	
MYH8	TTTCCACCAAGAACCCAGAG	CACTCATGGCTGCGATTTATTT	
MyoD	CTCCAACTGCTCCGACGGCAT	ACAGGCAGTCTAGGCTCGACAC	
Myogenin	AGTGCCATCCAGTACATCGAGC	AGGCGCTGTGAGAGCTGCATTC	
RPLP0	TGGTCATCCAGCAGGTGTTCGA	ACAGACACTGGCAACATTGCGG	
TBP	TGTATCCACAGTGAATCTTGGTTG	GGTTCGTGGCTCTCTTATCCTC	
TNNI2	ATGGGAGATGAGGAGAAGCG	GCAGCATCACACTCTTCAGG	
			
**Plasmids**	**Description**	**Source**	
Lenti-hCMV-KDM5A-eGFP-IRES-BSD	Lentiviral plasmid with KDM5A open reading frame	Custom design for this study, ordered at Transomic Technologies	
GFP-pLenti6/V5-DEST	Lentiviral plasmid with GFP open reading frame	Previously generated by Haidar et al., 2019	

Abbreviations: ICC: immunocytochemistry; HRP: horseradish peroxidase; qRT-PCR: quantitative Reverse Transcription Polymerase Chain Reaction

## Data Availability

Description: Extended Data file with 1) validation of KDM5A overexpression throughout differentiation, 2) raw and uncropped blots used in panel E.. Resource Type: Image. DOI: https://doi.org/10.22002/9j1nz-sc824
